# An Analysis of the Knowledge Among Midwifery Students at Medical University-Pleven Regarding Human Papillomavirus (HPV) and HPV-Associated Diseases

**DOI:** 10.7759/cureus.66154

**Published:** 2024-08-05

**Authors:** Elitsa Y Petkova, Mariela S Kamburova, Eleonora N Mineva-Dimitrova

**Affiliations:** 1 Faculty of Health Care, Department of Midwifery, Medical University – Pleven, Pleven, BGR; 2 Faculty of Public Health, Department of Social Medicine and Health Management, Medical University – Pleven, Pleven, BGR

**Keywords:** knowledge, female medical students, s: health literacy, human papillomavirus (hpv), midwife

## Abstract

Background and objective

Comprehensive health literacy and prevention have been the key methods to reduce the spread of human papillomavirus (HPV) and HPV-associated disease development. Raising awareness among young individuals about the risk factors and the ways to prevent the infection is often the starting point of primary prevention. In light of this, we aimed to assess the awareness of midwifery students at Medical University-Pleven about (HPV) and HPV-associated diseases.

Material and methods

We conducted a survey-based study among first-year students at Medical University-Pleven in the period spanning January to March 2020, which involved a direct group survey. We initially reached out to 445 students and 284 (63.8%) of them responded; 12 of them were midwifery students. In the period from May through November 2022, the same type of survey was repeated among 75 midwifery students, and 47 (62.7%) responded. A set of classic statistical methods were used to present and analyze the collected quantitative and qualitative data. The responses in the questionnaires were reviewed and recoded according to the requirements of the statistical program. The significance of the results, the findings, and the conclusions was set at p<0.05. A comparative analysis was employed to statistically compare the results to present the differences between the groups of traits studied. Data processing was performed using MS Office Excel 2019 and SPSS Statistics v.28 (IBM Corp., Armonk, NY).

Results

Over half (70.6%) of the first-year midwifery students were aware of the infection caused by HPV. Among them, 10 students (29.4%) were familiar with the risk factors for HPV and HPV-associated diseases, and all of the fourth-year respondents knew about the studied issue. The majority of the respondents - 61.8% of the freshmen and 100% of the fourth-year students- were aware of HPV vaccine availability.

Conclusions

In the course of their training, the midwifery students at Medical University-Pleven acquired enough knowledge about the risk factors of HPV-associated diseases and the availability of vaccines to prevent them.

## Introduction

The human papillomavirus (HPV) is considered the most common cause of sexually transmitted diseases. In a 2015 report on global surveillance of sexually transmitted infections, the WHO estimated that globally over 290 million sexually active females would become infected with HPV at a certain point in their lives [1]. In the same year (2015), a survey was carried out among 1050 females in Bulgaria, which revealed a high prevalence of type 16 HPV. As per the study data, the heavily affected districts were as follows: Burgas (51.2% of the surveyed females), Sofia (51%), Varna (47.3%), Plovdiv (45.6%), Vidin (41.7%), and Pleven (30%) [2]. In 2021, the Information Center on HPV and Cancer (ICO/IARC) in its report on HPV-associated diseases stated that 3.10 million females over the age of 15 in Bulgaria belonged to the risk group for the development of HPV-associated disease [3]. Prevention has been the main method to reduce the virus spread and the development of diseases associated with it. Raising awareness among young people about the risk factors and the ways to prevent the infection has been critical in primary prevention [4].

In 2016, Rashid et al. carried out a survey among students in India regarding their awareness and knowledge of HPV, HPV vaccines, and cervical cancer. The authors found the respondents’ awareness of HPV, HPV vaccines, and cervical cancer to be insufficient [5]. In 2019, Kasymova et al. reported that HPV remained a serious problem in the USA. The authors found a low level of awareness among their respondents and highlighted the need for additional educational initiatives about HPV [6]. In 2019, Cinar et al. surveyed 1,563 students at Pamukkale University in Turkey and concluded that Turkish students did not have sufficient knowledge about HPV infection [7]. Wang et al. carried out a study (2021) on the awareness and knowledge among secondary school students in occupational medicine in China. Survey data revealed low rates of HPV-related knowledge among respondents. The conclusions drawn by the authors endorsed the findings of Rashid et al. (2016), Kasymova et al. (2019), and Cinar et al. (2019) [‎5,6,‎7]. From May 2019 through June 2020, Yacouti et al. surveyed 1087 students from six Moroccan universities. The authors demonstrated the insufficient awareness of the respondents related to HPV and HPV vaccines [8].

An analysis of the relevant literature sources proved that low health literacy and lack of knowledge about HPV and its associated diseases has been a global problem, prompting the authors to conclude that secondary school students and medical university students worldwide had poor awareness and low healthcare-related knowledge regarding the studied group of diseases. Yacouti et al. concluded that various awareness programs needed to be implemented among females at a young age [8]. Aleksieva (2020) conducted a study related to sexual health education in schools, involving 36 midwives and 228 teachers from Sliven Region. According to both groups of respondents, sexual health was influenced by the family and school environment. Of note, 91.70% of the surveyed midwives felt prepared to pass on their knowledge to secondary school students on sexual health issues. The author concluded that these healthcare professionals had already acquired sufficient knowledge from medical universities [9].

The objective of the present study was to analyze the knowledge acquired by the midwifery students at Medical University-Pleven, regarding HPV and HPV-associated diseases during their training.

## Materials and methods

Study design

This study employed a sociological method and involved a group questionnaire. Two groups were included in the study: first-year students and fourth-year midwifery students. The midwifery specialty at the Medical University-Pleven is a four-year training program. Students gain knowledge about HPV and its associated diseases in the following disciplines:

Gynecology-special part - 4th semester; dermatology and venereal diseases - 5th semester; specialized care for gynecological diseases - 5th semester; midwifery and nursing care for oncologically ill women - 5th semester; specialized care for pregnant women, women in labor, and gynecological patients with infectious and venereal diseases - 6th semester.

The disciplines are included both in the obligatory training course in the curriculum and in the Unified State Requirements (USR) [10].

Participants

The study participants were midwifery students at the Medical University-Pleven, Bulgaria. From January through March 2020, a survey was carried out among 35 first-year students at Medical University-Pleven, through a direct group survey. From May through November 2022, the same type of survey was conducted among 25 fourth-year midwifery students.

Statistical analysis

Statistical analysis was carried out to determine the significance of the differences in the knowledge between the two groups of students. The chi-square test was employed to identify differences between the two groups and determine the relationship between variables by using Cramer's V. The significance of the results, the findings, and the conclusions was set at p<0.05. Data processing was performed using MS Office Excel 2019 and SPSS Statistics v.28 (IBM Corp., Armonk, NY).

## Results

**Figure 1:**
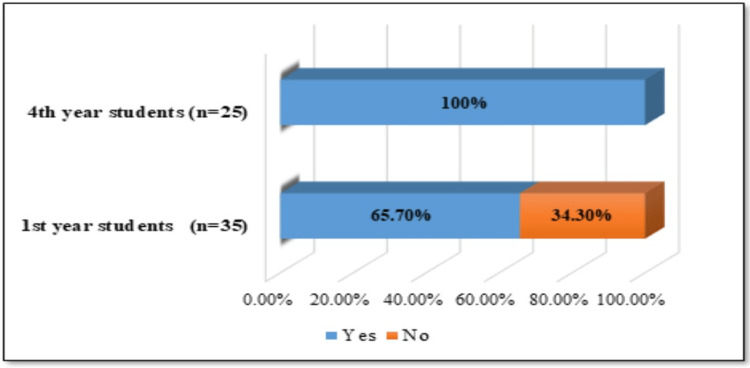
Students' knowledge of HPV infection (n=60) HPV: human papillomavirus

The study involved 60 midwifery students at the Medical University of Pleven. All participants were female. The results revealed that over half of the first-year students were familiar with the infection caused by HPV (n=23, 65.7%). On the other hand, 34.3% (n=12) of the respondents expressed a low level of awareness. A high level of knowledge about HPV infection was found (100.0%) among the surveyed fourth-year students. The data are presented graphically in Figure 1.

A statistically significant correlation was found between the respondents’ year of training and their awareness of HPV infection (χ2=10.714, df=1, p=0.01, Cramer's V=0.423).

**Figure 2:**
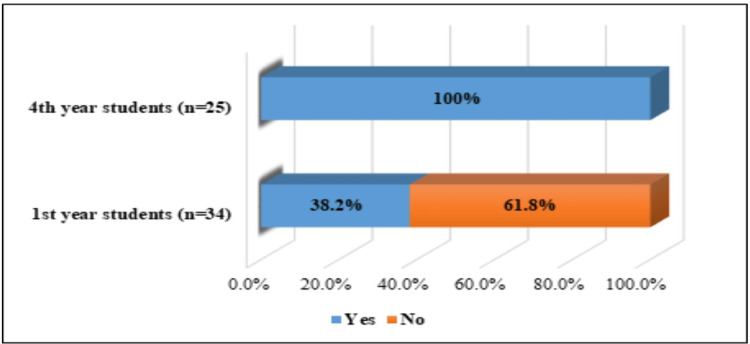
Students’ knowledge of risk factors of HPV infection HPV: human papillomavirus

The results demonstrated that among the surveyed midwifery students, at the beginning of their higher education, 13 (38.2%) were aware of the risk factors for HPV infection. Figure 2 presents the dynamics of knowledge at the beginning of the education and at the end of the training course; 25 (100.0%) of the fourth-year students showed a high level of health literacy related to the risk factors for HPV infection.

The main method used in the primary prevention of HPV-associated diseases is vaccination. The survey questionnaire contained six questions about the primary prevention of the studied group of diseases.

**Figure 3:**
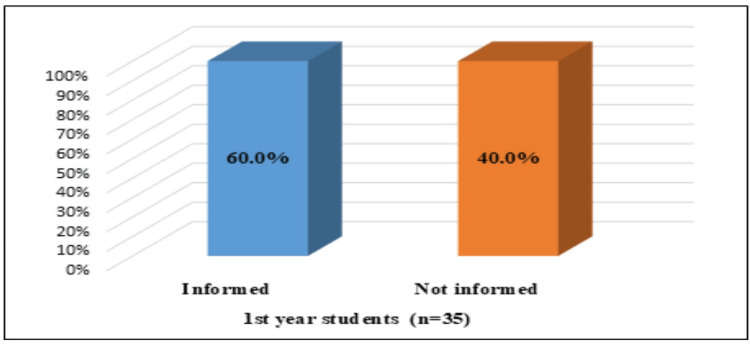
Awareness of first-year midwifery students about the existence of HPV vaccines HPV: human papillomavirus

Figure 3 shows the data for the first-year students, referring to their awareness of the availability of the HPV vaccine. Our findings revealed that the awareness rate among this group of respondents was slightly above the average level (21 students or 60.0%). Of note, 100.0% (25 students) of fourth-year midwifery students had good knowledge regarding the HPV vaccine.

The survey found a significant correlation between the awareness related to the existence of vaccines and the year of study of the students (χ2=13.043, df=1, p=0.00, Cramer's V=0.466).

**Figure 4:**
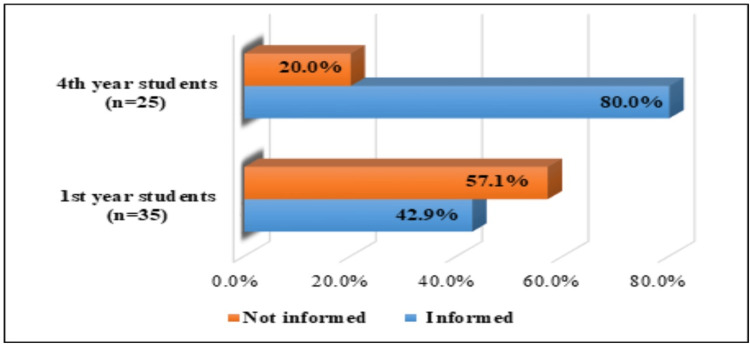
Awareness among first- and fourth-year midwifery students about the supply of HPV vaccines in Bulgaria HPV: human papillomavirus

The awareness rates of the first-year respondents, in relation to the supply of the HPV vaccine in Bulgaria, were as follows: 42.9% (15 female students) were informed, while over half of them were not informed (20 students or 57.1%). The fourth-year students showed a higher level of knowledge about the supply of the vaccine in the country, but their number did not reach 100.0% (25 students). Of them, 20 female students (80.0%) were informed. The data are graphically presented in Figure 4.

The immunization coverage among the respondents was of interest to the authors’ team. The survey found that among first-year female students, 24 (68.6%) were not vaccinated, and six (17.1%) stated that they did not know whether they had received the HPV vaccine or not. Among fourth-year students, 22 (88.0%) pointed they were not vaccinated. No one in this group knew whether they had been vaccinated against HPV. The respondents’ opinion was required regarding additional information about vaccines. 79.4% (27 students) of the freshmen and 84.0% (21 persons) of the group of fourth-year students stated that they wanted to receive more information about the preparations.

The respondents were asked for their opinion on the source of additional information they would use. The results showed that the largest segment of first-year students would seek information from university professors (14 students or 41.2%), while among fourth-year students, an equal number of respondents would turn to a midwife (nine students or 36.0% of the group) and a gynecologist (36.0% or nine students). The data thus showed that first-year students had a low rate of trust in the midwife as a person from whom they would like to receive additional information about vaccines (five students or 14.7%). That was because they were not yet familiar with the nature of a midwife’s work and the activities she could perform.

## Discussion

Our analysis of the obtained data revealed that first-year students majoring in midwifery at MU-Pleven, at the beginning phase of their higher education, were insufficiently informed about HPV and HPV-associated infection. The positive result in the fourth-year students revealed that university education promoted a culture of information regarding health-related matters. Regarding the risk factors for HPV infection, the low level of knowledge among the first-year students implied that the respondents did not know how to protect themselves. The results supported the conclusions by Simeonova in her dissertation thesis that education related to risky health behavior should be introduced in schools [11]. A similar conclusion was reached by Aleksieva, who stated that good sexual health was linked to early education about the same, which was also confirmed by the negative results among first-year students [9].

Insufficient awareness related to the risk factors for HPV infection would lead to high rates of morbidity among the young population. This was endorsed by the paper published by Kovachev et al. (2015), where the authors described that the highest rates of HPV prevalence were found in the age group 15-55 years [12]. First-year students were well informed about the existence of the HPV vaccine, but they had lower awareness about the variants available in Bulgaria. Even fourth-year students were not 100% knowledgeable in this aspect. In this group of respondents, only 75.0% (nine students) were aware of the variants supplied in our country. The data align with the conclusions drawn by Kostadinova et al. that insufficient awareness was the main reason for the low rates of HPV vaccination in Bulgaria [13], which was validated by the high ratio of unvaccinated students among the surveyed groups (75.0% of freshmen and 83.3% of fourth-year students).

Khatiwada et al. carried out a survey in 2019 among students in the specialties of medicine, nursing, and social sciences in Indonesia. The authors found that of the 372 respondents, 256 (68.8%) knew about HPV vaccines before participating in the study, while 116 (31.2%) had no information. The authors’ data confirmed our data as well as the conclusion that students had low awareness of HPV vaccination opportunities [2]. However, the results obtained by Cocchio et al. (2020) contrast with ours. The authors’ team carried out a survey at an Italian university from October 2015 to June 2016. They found that students had a good awareness of HPV vaccines (69.9%) [14]. Our results are endorsed by Lakneh et al. (2022), who found that 45.3% (281 individuals) of respondents had good knowledge related to the HPV vaccine [15].

The low confidence the first-year students expressed in the midwife as a specialist from whom they would like to receive additional information about vaccines was because they had not yet begun to study all the specifics of the competencies of this profession. This was further evidenced by the responses of the fourth-year students, who said they would seek information from a midwife (33.3%). At the end of their training, students will have gained knowledge about the overall specifics of a midwife’s work, as well as about independent activities and competencies.

## Conclusions

The study found that midwifery students at Medical University-Pleven acquired a high level of knowledge about HPV infection and the risk factors associated with the virus by the time they reached their fourth year. At the end of their training, students become highly health literate in general and specifically on issues related to HPV vaccination. They are aware that it is available all over the world and in Bulgaria. This study proved that training according to the curriculum was sufficient for acquiring the necessary knowledge related to the risk factors for HPV infection and HPV-associated disease development.
